# Supplementary material for: Molecular Drivers of RNA Phase Separation
Source: bioRxiv. 2025 Jan 22:2025.01.20.633842. Preprint. [Version 1] doi: 10.1101/2025.01.20.633842 (PMC11785085; doi:10.1101/2025.01.20.633842)
Supplement: Supplement 1 [file NIHPP2025.01.20.633842v1-supplement-1.pdf]

bioRxiv preprint doi: https://doi.org/10.1101/2025.01.20.633842; this version posted January 22, 2025. The copyright holder for this preprint (which was not certified by peer review) is the author/funder, who has granted bioRxiv a license to display the preprint in perpetuity. It is made available under aCC-BY 4.0 International license.

Supporting Information

Figure S1-S18
